# Shallow Groundwater Quality and Its Controlling Factors in the Su-Xi-Chang Region, Eastern China

**DOI:** 10.3390/ijerph17041267

**Published:** 2020-02-16

**Authors:** Jianwei Bu, Ziyong Sun, Rui Ma, Yunde Liu, Xulong Gong, Zhao Pan, Wenhao Wei

**Affiliations:** 1 Technology Innovation Center of Geo-Environmental Restoration, Ministry of Natural Resources, No. 388 Lumo Road, Wuhan 430074, China; jwbu@cug.edu.cn (J.B.); rma@cug.edu.cn (R.M.); 2 School of Environmental Studies, China University of Geosciences, No. 68 Jincheng Street, Wuhan 430078, China; lydcn84@126.com (Y.L.); panzhao@cug.edu.cn (Z.P.); 3 Key Laboratory of Earth Fissures Geological Disaster, Ministry of Natural Resources, No. 700 Zhujiang Road, Nanjing 210018, China; xulonggong@126.com; 4 Geological Survey, China University of Geosciences, No. 388 Lumo Road, Wuhan 430074, China; weiwenhao048@cug.edu.cn

**Keywords:** groundwater quality, multivariate statistics, hydrogeochemical processes, anthropogenic impacts, Su-Xi-Chang region

## Abstract

Understanding factors influencing groundwater quality is critical to the development of best management practices at the large watershed scale. In this study, the shallow groundwater (10–20 m depth) in the Su-Xi-Chang region, eastern China, was investigated as part of a monitoring program from 2007 to 2008 to analyze the regional groundwater quality as well as the hydrogeochemical processes and their controlling factors. Conventional physicochemical water parameters (pH, turbidity, electrical conductivity, dissolved oxygen, total phosphorus), major cations (Na^+^, Ca^2+^, Mg^2+^ and NH_4_^+^) and anions (Cl^−^, NO_3_^−^ and SO_4_^2−^) were measured. Hydrochemical methods and multivariate statistical methods were applied to analyze the hydrogeochemical signatures, origins, the similarities among the variables and to identify the main pollution sources in the groundwater. The results showed that (1) the concentrations of TDS (224.89–1086.70 mg/L) and turbidity (0.1–18.60 NTU) were higher than the class II groundwater quality standards in China and the WHO drinking water standards, (2) there were extremely high concentrations of ammonia (0.01–32.90 mg/L), with a mean value of 0.72 mg/L and (3) the nitrate concentrations (average value of 22.07 mg/L) exceeded the class III groundwater quality standards. The study also provided evidence that weathering, dissolution of carbonate, halite and silicate and cation exchange were the possible primary hydrogeochemical control mechanisms in the groundwater. The sources of ammonia, total phosphorus, sulfates and nitrates included rock–water interactions and anthropogenic activities. The groundwater administration of pollution sinks and sources, long-term legal frameworks and economic incentives should be improved to optimize watershed scale management in the context of rapid development in China.

## 1. Introduction

The water crisis is one of the most serious problems in the 21st century which is in fact continuing to get worse due to anthropogenic activities [1,2,3,4]. These activities involve the discharge of increasing amounts of contaminants into water bodies and excessive development and utilization of surface water and groundwater resources [3,5,6,7,8,9]. Owing to rapid urbanization, industrialization and intensified land reclamation, surface water has become destabilized, degraded and contaminated worldwide, which makes the availability and quality of groundwater, particularly shallow groundwater (0~60 m), an environmental concern [10,11,12].

Shallow groundwater is the major source of potable, domestic and industrial water throughout the world because in the absence of surface water, developing and utilizing groundwater is the compelling option. Moreover, where groundwater is available, obtaining shallow groundwater is more economical and convenient than deep groundwater [13,14,15]. However, the shallow groundwater has direct recharging and discharging relationships with local precipitation and surface water and is greatly affected by anthropogenic activities due to shallow buried conditions and rapid circulation [16,17,18]. Therefore, the quality and safety of shallow groundwater have drawn global attention. Investigating the spatial and temporal variation of shallow groundwater quality at a large scale is particularly useful for developing an effective water management system [19,20,21].

The Su-Xi-Chang region (including the municipalities of Suzhou, Wuxi and Changzhou) is located in the lower reaches of the Yangtze River in the southern Jiangsu Province. Sitting on the Yangtze River estuary and adjacent to Shanghai, the Su-Xi-Chang region is one of the fastest growing, most prosperous and representative regions in China. In this region, surface water and groundwater have frequent and intense interactions. Meanwhile, the resources of surface water and groundwater are abundant. Due to the shallow burial of shallow groundwater, the aquifer is easily polluted by the sewage discharged from factories and pesticide residues from farmland, which may also inversely influence the surface water body (e.g., Yangtze River, Taihu Lake, East Sea and the Yellow Sea) horizontally and deep groundwater vertically. Hence, it is extremely important to carry out research on shallow groundwater in areas where anthropogenic activities are frequent and the economy is developed, such as the Su-Xi-Chang region. However, few scientific, systematic and long-term groundwater measuring or monitoring programs have been conducted in this region in recent decades. Many studies regarding the occurrence of land subsidence due to excessive groundwater exploration have been performed in local areas [22,23,24,25]. Nevertheless, the hydrochemical characteristics, groundwater formation mechanisms and anthropogenic impacts on groundwater quality due to rapid urbanization and industrialization are less commonly reported. 

During the past decade, several approaches have been developed to assess the impacts of anthropogenic activities on water quality. There are two main obstacles to overcome. First, adequate long-term measurements of water quality data are even scarcer than data on water fluxes. Second, the transformation of small-scale process descriptions to the watershed or catchment scale remains an unsolved problem [26]. Multivariate statistical methods can be used to characterize and evaluate the temporal and spatial variations of groundwater quality from complicated datasets [11,27,28,29,30]. Spatially referenced regressions of contaminant transport in watershed attribute models have been used as a hybrid process-based and statistical modeling approach for estimating pollutant sources and contaminant transport in waters [31,32]. Recent studies in large watersheds that have utilized remote sensing data and geographical information techniques have been oriented toward quantifying the combined influence of watershed characteristics, land-use changes and social and economic factors on nutrient discharge into rivers [21,30,33,34,35,36].

The objectives of the present study were to (1) detect the conventional physicochemical parameters (pH, turbidity, electric conductivity, dissolved oxygen, total phosphorus) of the groundwater, (2) determine the concentrations of major cations (Na^+^, Ca^2+^, Mg^2+^ and NH_4_^+^) and anions (Cl^−^, NO_3_‑ and SO_4_^2−^) in the groundwater, (3) analyze the hydrogeochemical signatures, processes, origins and mechanisms in the study area and (4) identify the anthropogenic impacts on groundwater quality variations in the Su-Xi-Chang region in eastern China.

## 2. Outline of Study Area and Methodology

### 2.1. Study Area

**Figure 1:**
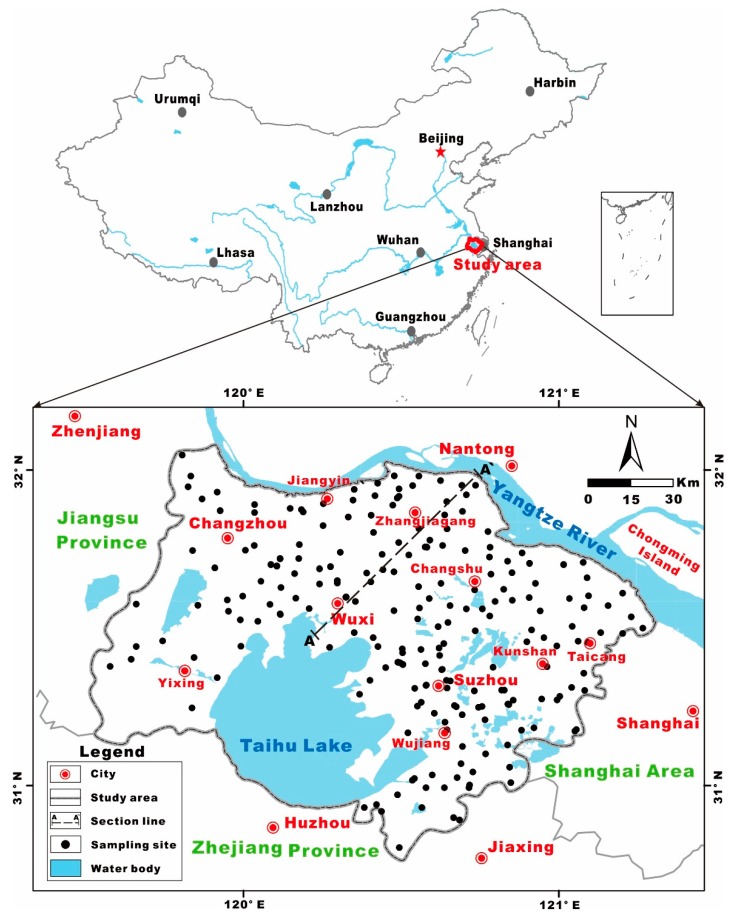
The geographic location of the Su-Xi-Chang region with the sampling sites.

The Su-Xi-Chang region is situated in the Taihu Lake plain in the Southern Yangtze Delta in the eastern part of China and constitutes approximately 14,649 km^2^ of the territory; additionally, the average elevation is below 50 m (decreases from the west to the east), as illustrated in Figure 1. It borders the Mogan Mountain and Maodong Plain in the west, the East Sea and the Yellow Sea in the east, the Yangtze River in the north and Zhejiang Province in the south [23]. The northern subtropical monsoon climate dominates this region year-round, with an average annual temperature of 17.5 °C and a mean annual precipitation of 1055 mm, all of which are beneficial for hydrological cycling and hydrogeochemical transformation [37].

### 2.2. Hydrogeological Conditions

**Figure 2:**
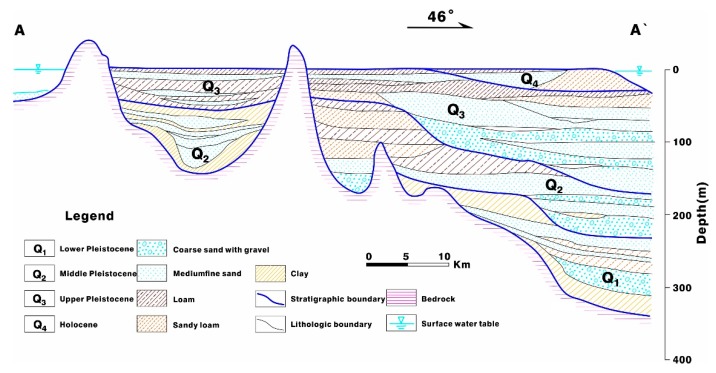
Hydrogeological profile of the Quaternary phreatic and confined aquifers.

Various groundwater types, complex buried conditions and uneven spatial distributions of groundwater were observed in the study area, which clearly indicates the regional characteristics [22,24,38]. Pore water in unconsolidated materials is the primary type of groundwater in the plain area. According to the origins, ages, buried distributions, hydraulic connections and hydrochemical characteristics of the aquifers, they were divided into phreatic aquifers and the confined aquifers in the area were labeled from top to bottom as I, II and III [23,25]. The soil intervals between the aquifers are viscous and the lithology is dominated by loam and sandy loam with medium-fine sand, which were regarded as lenses for aquitard or aquifuge (Figure 2).

The pore-phreatic aquifer, which is the target aquifer in this study, consisting of Holocene and Upper Pleistocene, loam, sandy loam and silty sand, is distributed throughout the study area. Although the phreatic groundwater quality is complicated, freshwater mostly occupies the area. From the west to the east, the hydrochemical types of phreatic groundwater vary from the HCO_3_—Ca·Mg type to the Cl·HCO_3_—Na type.

Confined aquifer I, which is composed of Upper Pleistocene loam, sandy loam, silty sand and medium-fine sand, is characterized by a multi-layered structure with mostly HCO_3_-Na·Ca-type groundwater.

As the main groundwater exploitation layer in the study area, confined aquifer II is composed of 1–3 layers of Middle Pleistocene loam, sandy loam, fine sand, medium coarse sand, pebbly coarse sand and gravel. Due to the stability (mostly HCO_3_—Na·Ca type) and excellence (mineralization 0.60–0.90 g/L) of the groundwater quality, confined aquifer II is suitable as a direct drinking water source.

Confined aquifer III consists of clay, sandy loam, sand and gravel. The quality of the groundwater in this aquifer is considered to be good and is accompanied by HCO_3_—Ca·Na and HCO_3_·Cl—Na·Ca hydrochemical types and a mineralization of 0.75–0.90 g/L.

### 2.3. Sampling and Analytical Methods

A total of 231 shallow groundwater samples (10~20 m in depth) were collected throughout the study area as part of a groundwater monitoring program based on existing wells, springs, newly built monitoring wells and boreholes from December 2007 to January 2008. The layout of the high-density sampling sites generally ensured uniformity and representativeness. The elevation and coordinates of the sampling sites were measured and identified using a portable handheld global positioning system (GPS).

Physicochemical parameters were measured at each sampling site and all samples were analyzed for major cations, anions and alkalinity. Water temperature, pH, turbidity, dissolved oxygen (DO) and electric conductivity (EC) were measured in the field at the time of collection using a digital handheld multiparameter sampling instrument (YSI ProDSS) [3]. The samples were pretreated during field collection using a degassing filtration system (GM-0.33A, JINTENG) powered by vehicular electricity supply, accompanied with a 0.45 μm water-soluble filter membrane and acidified to pH ≤ 2 using ultra-pure HNO_3_ for the cation analysis [39,40]. Without the determination of NOM (Natural Organic Matter) or TOC (Total Organic Carbon), such on-site pretreatment can effectively filter the particle impurities, suspended solids and complexes, and stabilize the cations in the water sample, avoiding subsequent disturbance so as to make the measured results of cations and anions more accurate and the instruments more reliable. After sample collection, each sample was stored in polyethylene bottles that were sealed with parafilm sealing film before further chemical analysis at the State Key Laboratory of Biogeology and Environmental Geology, China University of Geosciences (Wuhan, China).

Major cations (Na^+^, Mg^2+^ and Ca^2+^) were determined by inductively coupled plasma optical emission spectrometry (ICP-OES) (iCAP 6300, Thermo Fisher Scientific) on the filtered and acidified samples. The average analytical error of the major and trace chemical constituents using ICP-OES is less than ±5%. The major anions (Cl^−^, NO_3_‑ and SO_4_^2−^) were detected by ion chromatography (IC) (ICS 1100, DIONEX) with a detection limit of 0.01 mg/L. Alkalinity was measured by in situ titration with HCl (0.1 N), using methyl orange as an indicator within 6 h of sampling. Total phosphorus (TP) was determined by the colorimetric method after previous digestion [3] and the absorbance readings together with the ammonia were performed in a spectrophotometer (1600 PC, Shimadzu).

The ionic balance error was within ±5%, as the percentage relative total of the cation–anion difference was calculated on the sums from each groundwater sample. All analyses yielded analytical errors <5% and external precision of known–unknown analytical standards. All procedures of sampling, preservation and transportation to the laboratory were conducted according to standard methods [41].

### 2.4. Data Treatment and Analytical Methods

Multivariate statistical methods, including factor analysis (FA), cluster analysis (CA) and principal component analysis (PCA) were used to analyze the variation in water quality and its relationship with anthropogenic descriptors [21]. PCA is a widely used technique to reduce the dimensions of multivariate data and explain the correlations among large numbers of observed variables by extracting a smaller number of latent factors (i.e., principal components or PCs) [35,42,43,44]. Eigenvalues represent the amount of variance explained by the data matrix. Loadings represent the relative importance of a given variable in a given component. A positive or negative loading means that the variable is positively or negatively correlated to the component [21]. To improve the interpretability of the results, a PCA with varimax normalized rotation was applied; this can maximize the variances in the loading factor across the variables of each factor [45]. In this study, three principal factors extracted from the variables were retained with eigenvalues greater than 1.0, as determined by the Kaiser criterion [46]. Descriptive statistics, correlation coefficients and PCA were all performed using SPSS Version 19.0.

## 3. Results and Discussion

### 3.1. Physicochemical Parameters, Hydrochemical Characteristics and Groundwater Quality

A statistical summary of the physicochemical parameters of the shallow groundwater in the Su-Xi-Chang region is given in Table 1. To assess the suitability of the shallow groundwater over the study area for domestic purposes, the groundwater samples were compared with quality standards for groundwater (GB/T 14848-93 [47]), standards for drinking water quality (GB 5749-2006 [48]) and drinking water quality guidelines [49]. For effective disinfection with chlorine, the pH should preferably be less than 8; however, lower-pH water (approximately pH 7 or less) is more likely to be corrosive [49]. In this study, the pH in the shallow groundwater was observed within the range from 5.59 to 7.82 with a mean value of 7.0, which indicated that the groundwater was faintly acidic to mildly alkaline and well within the permissible limit of 6.5–8.5 [48,49]. Total dissolved solids (TDS) is an important indicator of drinking water quality. According to the World Health Organization (WHO) and the Ministry of Health (MH) of China, the palatability of water with a TDS level of less than approximately 600 mg/L is generally considered to be good; drinking water becomes significantly and increasingly unpalatable as the TDS levels reach greater than approximately 1000 mg/L [48,49]. In the present study, the TDS levels of the groundwater ranged from 224.89 to 1086.70 mg/L, with an average value of 616.69 mg/L. However, the average TDS level was still higher than the quality standard limits for class II (≤500 mg/L) groundwater established by the State Bureau of Technical Supervision (GB/T 14848-93 [47]). Within this class II limit, the groundwater is recommended to be used for a variety of purposes. Approximately 74% of the samples in the study area were at TDS levels greater than 500 mg/L.

Both turbidity and dissolved oxygen (DO) are important parameters that reflect the quality of groundwater. The turbidity was measured in the range from 0.1 to 18.60 NTU, with a mean value of 3.85 in all samples. According to the quality standards for class II groundwater, the turbidity should be less than 3 NTU (GB/T 14848-93 [47]), although this limit is even stricter for drinking water usage [48,49]. Only 30% of the groundwater samples qualified for drinking condition in terms of turbidity (≤1 NTU), while 40% of the samples failed to reach the class II groundwater conditions. Despite the fact that DO is not specified in either groundwater standards or drinking water standards, it is critical to the survival of aquatic organisms. The mean DO value was only 0.97 mg/L, which was considered low in the study area.

In water bodies, ammonia and total phosphorus (TP) are important parameters indicative of inorganic pollution that mainly result from anthropogenic impacts. They were used as the primary restrictive descriptors of water pollution control goals in the 12th Five-Year Plan of China (2011–2015) [21]. The reference ammonia concentration value established by the China State Bureau of Technical Supervision (CSBTS) for class II groundwater is 0.02 mg/L. However, the average ammonia value reached 0.72 mg/L in this study and the maximum value was 32.90 mg/L. Some sampling sites are located in urban areas and are presumably influenced by industrial effluent or domestic sewage discharge, which may contribute to the increased ammonia levels at these points. Although TP is not specified in groundwater or drinking water standards, the maximum and mean values are 2.02 and 0.15 mg/L, respectively, which are high compared with the environmental quality standards for surface water (GB 3838-2002 [50]). The increase in ammonia and phosphorus levels at most sampling sites in strictly rural areas may be attributed to excreta from poultry and livestock and agricultural fertilizers used in the cultivation of farm crops, e.g., soybeans and corn, which are common in the study area.

As shown in Table 2, the major cations and anions in the groundwater show significant spatial variation in the study area. The concentrations of Ca^2+^, Mg^2+^ and Na^+^ in the groundwater over the study area were in the ranges of 26.20–162.00, 6.07–64.00 and 8.32–159.00 mg/L, respectively. The concentrations of HCO_3_^−^, Cl^−^ and SO_4_^2−^ in the groundwater were observed in the ranges of 113.00–706.00, 7.96–191.00 and 2.45–187.00 mg/L, respectively. More than 98% of all samples in the study area were within the permissible limits of major cations and anions for drinking water due to the TDS values ≤1000 mg/L (Table 1), excluding only 4 samples with their average TDS value of 1040.30 mg/L. According to the mean values, the major cations and anions in the groundwater in the study area were found in the order of Ca^2+^ > Na^+^ > Mg^2+^ and HCO_3_^−^ > SO_4_^2−^ > Cl^−^, respectively.

Groundwater nitrate contamination is currently very common and has become a growing problem in many regions of the world. Many researchers have reported serious groundwater nitrate pollution in China [51,52,53,54,55]. In the shallow groundwater in the Su-Xi-Chang region, NO_3_^−^ showed a wide concentration variation from 0.01 to 154.00 mg/L, with an average of 22.07 mg/L. Approximately 14% of the shallow groundwater samples have NO_3_^−^ concentrations greater than 50 mg/L, which is the WHO recommended maximum acceptable guideline value for drinking water [49]. These high concentrations can be attributed to the leaching of NO_3_^−^ from frequent anthropogenic activities such as fertilization and irrigation of agricultural land and wastewater leakage.

### 3.2. Hydrochemical Origin and Correlation Analysis

Gibbs diagrams have been proposed to investigate the mechanisms controlling the surface water chemistry throughout the world. Gibbs diagrams are constructed by plotting the ratios of Na/(Na+Ca) and Cl/(Cl + HCO_3_) weights versus TDS separately on a logarithmic axis. Based on extensive water chemistry data from numerous rain, river, lake and ocean samples, Gibbs diagrams have been classified into three dominance domains corresponding to precipitation dominance, rock dominance and evaporation dominance. In recent years, Gibbs diagrams have also been used to evaluate groundwater chemistry. Therefore, in this study, the samples were plotted on Gibbs diagrams to investigate the factors governing the chemistry of the shallow groundwater in the Su-Xi-Chang region.

**Figure 3:**
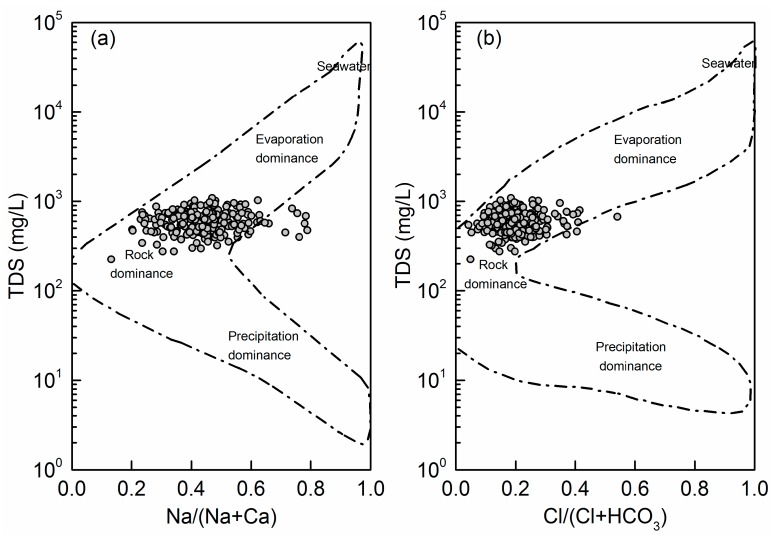
Gibbs diagrams for the shallow groundwater in the Su-Xi-Chang region.

As shown in Figure 3, all samples were distributed in the middle part (mid-range TDS levels) of the boomerang on the Gibbs diagrams, while Na/(Na+Ca) values spanned the entire range (0.1–0.8), which reflected the characteristics of fresh groundwater, unlike most surface water [56]. Almost every sample dropped in the shape of boomerang except for only a few dots with higher Na/(Na+Ca) values than 0.7. It can be inferred that carbonate minerals dominate the groundwater chemistry, accompanying the availability and solubility of silicates minerals [57]. Depending on soil and aquifer properties, all samples fell toward the rock dominance domain, suggesting that rock–water interactions are the major source of dissolved ions in the groundwater in this study.

To further understand the hydrogeochemical processes regulating the groundwater quality, a correlation analysis was performed and a bivariate diagram was constructed [58]. Pearson correlation (r) was used to examine the correlation between all possible variable pairs. Samples showing r > 0.7 are considered to be strong, whereas 0.7 > r > 0.5 represents moderate correlation at a significance level (*p*) of <0.05 (Table 3) [59,60].

As shown in Table 3, TDS was strongly correlated with Ca^2+^, Na^+^ and HCO_3_^−^ (r > 0.7) and moderately correlated with Mg^2+^, Cl^−^ and SO_4_^2−^, which indicates that the groundwater salinities were mainly controlled by these ions due to the dissolution of minerals or concentration by evaporation [61,62].

HCO_3_^−^ was related to Ca^2+^ and Mg^2+^ with high correlation coefficients (Table 3), which suggests the possibility of carbonate weathering and dissolution [63,64]. Generally, weathering and dissolution of carbonates such as calcite and dolomite during irrigation, rainfall infiltration and groundwater movement are the major sources of Ca^2+^, Mg^2+^ and HCO_3_^−^ as follows:

CaCO_3_ + H_2_O + CO_2_ → Ca^2+^ + 2HCO_3_^−^ (1)

CaMg(CO_3_)_2_ + 2H_2_O + 2CO_2_→ Ca^2+^ + Mg^2+^ + 4HCO_3_^−^ (2)

CaCO_3_ + H^+^ → Ca^2+^ + HCO_3_^−^ (3)

CaMg(CO_3_)_2_ + 2H^+^ → Ca^2+^ + Mg^2+^ + 2HCO_3_^−^ (4)

**Figure 4:**
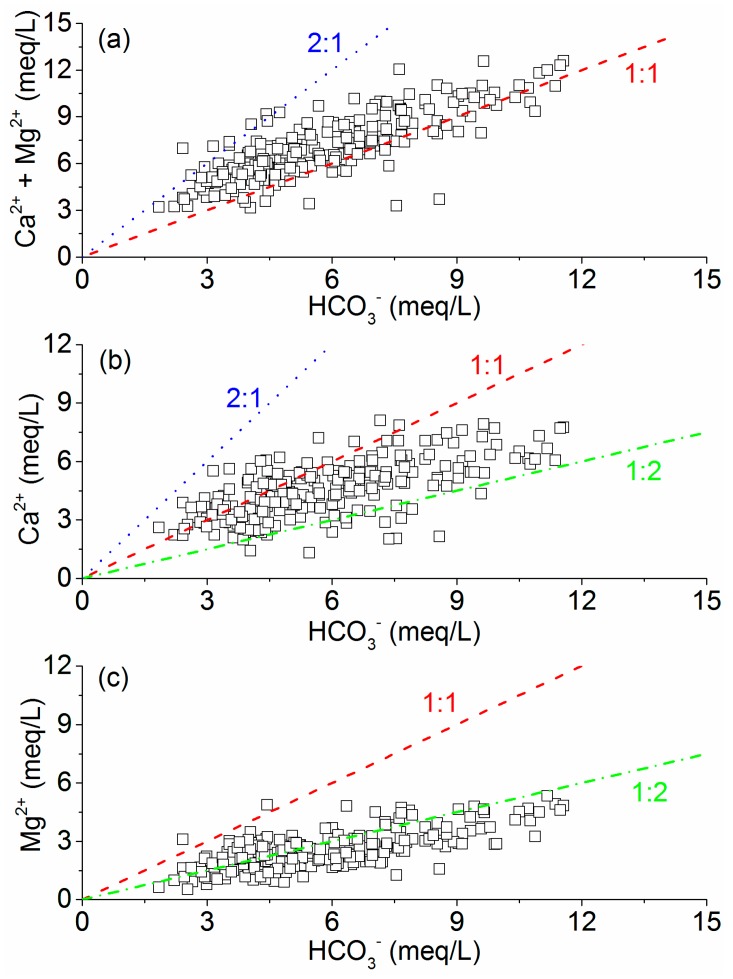
Bivariate diagrams of carbonate weathering and dissolution (**a**), calcite and dolomite weathering and dissolution (**b**,**c**).

Accordingly, the equivalent (Ca^2+^ + Mg^2+^)/HCO_3_^−^ ratio (expressed in meq/L) would be 1:1 to 2:1 if Ca^2+^, Mg^2+^ and HCO_3_^−^ in the groundwater originated solely from carbonate weathering [65]. Figure 4a indicates that carbonate weathering is a dominant hydrogeochemical process that controls the evolution of Ca^2+^, Mg^2+^ and HCO_3_^−^ in shallow groundwater. Some samples falling below the 1:1 line (Figure 4a) showed a depletion of Ca^2+^+Mg^2+^ relative to HCO_3_^−^ that suggested other hydrogeochemical processes, such as silicate weathering and/or cation-exchange processes, in the study area. Theoretically, if Ca^2+^ and HCO_3_^−^ in the groundwater originated solely from calcite weathering, the equivalent Ca^2+^/HCO_3_^−^ ratio would be 1:1 to 2:1 depending on the relative contribution of carbonic acid and strong acid (sulfuric acid, nitric acid), whereas this ratio is 1:2 to 1:1 for dolomite weathering. Similarly, the equivalent Mg^2+^/HCO_3_^−^ ratio is 1:2 to 1:1 for dolomite weathering depending on the relative contributions of carbonic acid and strong acid, whereas for calcite weathering, this ratio is 0 to 1:2. In the plot of Ca^2+^ versus HCO_3_^−^ (Figure 4b), a majority of the samples follow the 1:1 and 1:2 lines, indicating the contribution of both calcite and dolomite weathering to the groundwater chemistry in the study area, which is further supported by Figure 4c.

**Figure 5:**
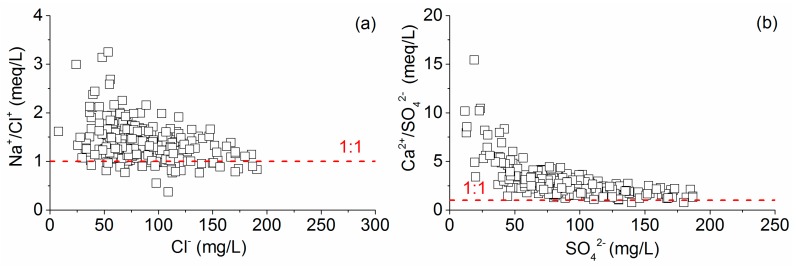
Bivariate diagrams of halite (**a**) and gypsum/anhydrite (**b**) weathering and dissolution.

Na^+^ usually comes from halite (NaCl) dissolution and silicate comes from albite weathering. Furthermore, cation exchange may also be responsible for the increase in Na^+^ concentration in groundwater. The influence of the controlled dissolution of halite on the concentrations of Na^+^ and Cl^−^ was suggested by a strongly correlated relationship (r = 0.706) between Na^+^ versus Cl^−^ concentrations (Table 3). The dissolution of halite will release equal amounts of Na^+^ and Cl^−^ into the groundwater. In other words, the equivalent Na^+^/Cl^−^ ratio would be 1:1 if halite dissolution is the sole source of Na^+^ and Cl^−^. However, higher Na^+^ concentrations with respect to Cl^−^ than expected from the theoretical 1:1 halite dissolution line were found in most of the groundwater samples (Figure 5a). The equivalent Na^+^/Cl^−^ ratio greater than 1 suggests that the groundwater underwent other processes such as silicate weathering (e.g., albite) as well as cation exchange rather than only the halite dissolution process, specifically in those samples with low Cl^−^ concentrations.

Similarly, the dissolution of gypsum and anhydrite will release equal amounts of Ca^2+^ and SO_4_^2−^ into the groundwater, which means that the equivalent Ca^2+^/SO_4_^2−^ ratio would be 1:1 if the dissolution of gypsum and anhydrite is the sole source of Ca^2+^ and SO_4_^2−^. In the study area, the dominant source of Ca^2+^ provided by gypsum and anhydrite dissolution and that of Na^+^ provided by the dissolution of the Na_2_SO_4_ minerals mirabilite and the nardite can be deduced from the highly significant correlations between Ca^2+^ versus SO_4_^2−^ and Na^+^ versus SO_4_^2−^ (Table 3). The equivalent Ca^2+^/SO_4_^2−^ ratio of the groundwater samples usually falls on or above the theoretical 1:1 line of gypsum and anhydrite dissolution and progressively reaches the gypsum and anhydrite dissolution line as the SO_4_^2−^ concentrations increase (Figure 5b). This result demonstrated that gypsum dissolution might be a contributing factor to Ca^2+^ and SO_4_^2−^ in the groundwater, especially in the groundwater with high SO_4_^2−^ concentrations. The excess of Ca^2+^ over SO_4_^2−^ resulted from the dissolution of carbonates such as calcite and dolomite, which will release Ca^2+^ into the groundwater but will not release SO_4_^2−^. In addition, reverse cation exchange may also increase the concentration of Ca^2+^ in the groundwater, in which Ca^2+^ adsorbed in the sediments is exchanged with Na^+^ in the solution.

**Figure 6:**
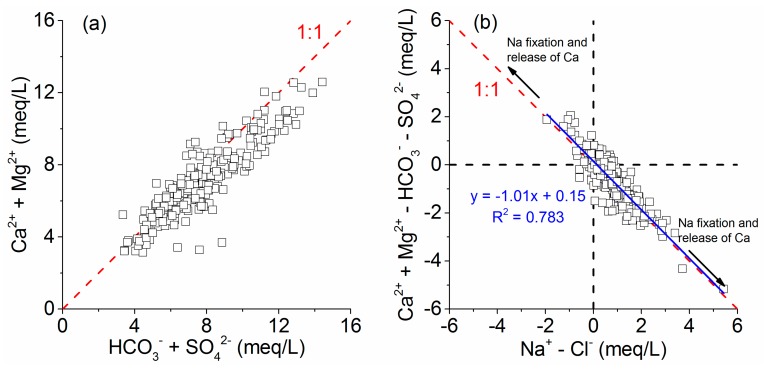
Multivariate diagrams of carbonate and sulfate weathering and dissolution (**a**), cation exchange (**b**).

The relationship between Ca^2+^+Mg^2+^ and HCO_3_^−^+SO_4_^2−^ indicates the contribution of the dissolution of carbonates (such as calcite and dolomite) and sulfate minerals (such as anhydrite and gypsum) to the groundwater chemistry [66]. If the dissolution of carbonates and sulfate minerals is the dominant hydrogeochemical process in the groundwater, the (Ca^2+^+Mg^2+^) to (HCO_3_^−^+SO_4_^2−^) stoichiometric ratios in meq/L of the samples should fall along the 1:1 line. However, most groundwater samples in this study showed a deficiency of (Ca^2+^+Mg^2+^) relative to (HCO_3_^−^+SO_4_^2−^) (Figure 6a). This result indicates that these ions were also regulated by other processes rather than only the dissolution of carbonates and sulfate minerals. The excess negative charge of (HCO_3_^−^+SO_4_^2−^) must be balanced by additional Na^+^ derived from the normal cation exchange process in the clay/weathered layer as well as silicate weathering. Although the excess (Ca^2+^+Mg^2+^) over (HCO^3−^+SO_4_^2−^) in a few samples indicates reverse cation exchange, the extent is much less. Furthermore, when cation exchange is a significant geochemical process for controlling the composition of groundwater, the relationship between (Ca^2+^+Mg^2+^-HCO_3_^−^-SO_4_^2−^) and (Na^+^-Cl^−^) should be linear with a slope of approximately −1 [67,68]. All the groundwater samples from the study area define a straight line (R^2^ = 0.78) with a slope of −1.01 (Figure 6b), which indicates that the cations including Na^+^, Ca^2+^ and Mg^2+^ participate in the ion exchange in the study area. It is also noteworthy that most samples in the study area plot within the fourth quadrant (negative ordinate and positive abscissa). This implies that there is abnormal cation exchange between Ca^2+^ or Mg^2+^ in the groundwater and Na^+^ and K^+^ in the aquifer material. In other words, a normal cation exchange reaction may be considered the dominant process that would explain an increase in Na^+^ related to a decrease in (Ca^2+^ + Mg^2+^). Furthermore, some samples also plot within the second quadrant (positive ordinate and negative abscissa), indicating reverse cation exchange.

In addition, the weathering of silicate minerals may be another dominant process in the study area, as shown by the dissolved silica data in Table 2. The weathering of albite and/or K-feldspar (Equations (5) and (6)) may also be responsible for the contribution of Na^+^ and/or K^+^ into the groundwater with the equivalent HCO_3_^−^.

2NaAlSi_3_O_8_+2CO_2_+11H_2_O → 2Na^+^+ 2HCO_3_^−^+4H_4_SiO_4_+ Al_2_Si_2_O_5_(OH)_4_ (5)

2KAlSi_3_O_8_ + 2CO_2_+ 11H_2_O → 2K^+^+ 2HCO_3_^−^+4H_4_SiO_4_+ Al_2_Si_2_O_5_(OH)_4_ (6)

Thus, the samples plotting within the fourth quadrant (negative ordinate and positive abscissa) in the bivariate diagram of (Ca^2+^+Mg^2+^-HCO_3_^−^-SO_4_^2−^) and (Na-Cl^−^) may also result from the weathering of soda feldspar (albite) and/or potash feldspars (Figure 6b). The dissolved silica data and the data related to K^+^ and Na^+^ apparently support the explanation that silicate weathering influences the water chemistry in the study area (Table 2 and Table 3) [67,68].

### 3.3. Principal Component Analysis

The hydrogeochemical datasets of the 231 groundwater samples were subject to multivariate analysis using principal component analysis (PCA). Table 4 displays the PCA results and the factor loadings of the shallow groundwater in the Su-Xi-Chang region with varimax rotation. Three PCs (FA1, FA2 and FA3) with eigenvalues greater than one [46] were extracted and represented 73.11% of the total variance in the hydrochemistry.

FA1 was responsible for 42.18% of the total variance and had highly positive loadings of 0.880, 0.823, 0.740, 0.682, 0.879 and 0.676 in TDS, major cations (Mg^2+^, Na^+^, Ca^2+^) and anions (HCO_3_^−^, Cl^−^), respectively. TDS, Ca^2+^, Mg^2+^ and HCO_3_^−^ are strongly associated, which represents carbonate weathering and ion exchange, as suggested by the correlation analysis. The high affiliation among TDS, Na^+^ and Cl^−^ may be attributed to halite dissolution. This factor is taken as a natural factor of hydrogeochemical processes and origins.

FA2 accounts for 18.85% of the total variance and is positively correlated with SO_4_^2−^ and NO_3_^−^, displaying high loadings of 0.756 and 0.820, respectively. This factor may be attributed to acid rain and human activities (irrigation, fertilizer and manure, domestic sewage, etc.). This result is supported by the fact that significant domestic and agricultural activities, as well as acid deposition due to rapid industrialization, occur in this region [69]. This is a conspicuous factor of anthropogenic impacts that can be confirmed by the aforementioned analysis.

FA3 is dominated by H_2_SiO_3_ with a high loading of 0.948 and accounts for 12.08% of the total variance. The highly positive correlation between FA3 and H_2_SiO_3_ can be explained by the process of silicate mineral weathering, which may also be responsible for the cations and anions, as supported by the correlation analysis shown in Table 3. FA3 can be treated as a special factor of hydrogeochemical process and behavior.

The sum of the squared factor loadings for all factors for a given hydrogeochemical parameter (row) is the variance in the variable that is accounted for by all factors and this is called the communality [70]. The communality measures the percent of the variance in a given hydrogeochemical parameter jointly explained by all factors and may be interpreted as the reliability of the indicator. To obtain the variance percentage in a given hydrogeochemical parameter that is accounted for by each factor, the squared factor loadings of the given hydrogeochemical parameter for that factor were divided by the corresponding communality. As shown in Table 5, the variability in TDS may be due to the effects of minerals weathering, dissolution and ion exchange, which can explain 80% of the three factors. For Ca^2+^, Mg^2+^, HCO_3_^−^, Na^+^ and Cl^−^, the contribution from carbonate weathering, halite dissolution and ion exchange can represent 78% to 94% of the three factors. The effects of acid rain and/or human activities (FA2) that were attributed to SO_4_^2−^ and NO_3_^−^ were greater than 80% relative to the other processes included in FA1 and FA3. The processes of silicate mineral weathering, dissolution, reaction, transportation and transformation in the groundwater can be divided from the main major hydrogeochemical processes given that H_2_SiO_3_ in FA3 dominated 99% of the three factors.

### 3.4. Anthropogenic Activities and Impacts

#### 3.4.1. The Status of Groundwater Exploitation and Utilization

**Figure 7:**
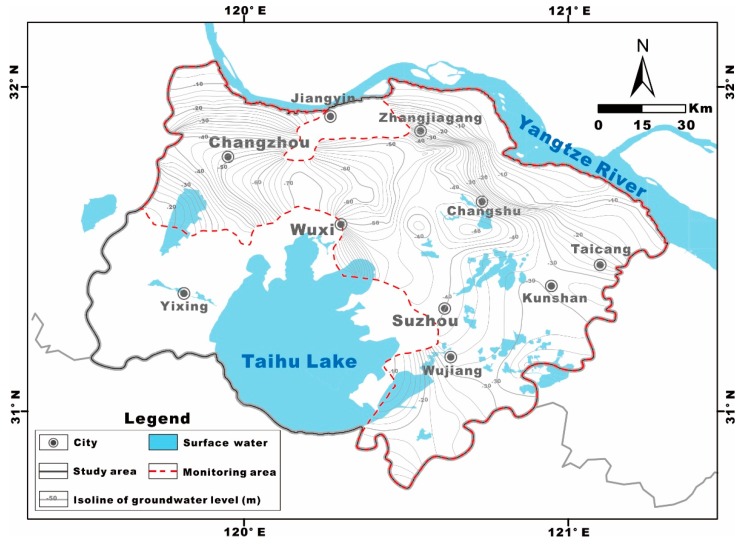
Contour plot of the groundwater table in the Su-Xi-Chang region (2015).

The exploitation and utilization of porous groundwater in the Su-Xi-Chang region have long histories. As early as the Warring States period more than 2000 years ago, the residents began to excavate the indigenous shaft for daily life usage with a depth of approximately 5 m. According to the survey, there are 4237 deep wells in the study area, with an annual exploitation quantity of 3.37 × 10^8^ m^3^, which equals 9.25 × 10^5^ m^3^ average daily extraction of groundwater. The exploitation of groundwater has already caused more than 10,000 km^2^ of regional groundwater drawdown cone and the groundwater in the center of the funnel has been drained (Figure 7).

From the view of exploited horizons, confined aquifer II is the main source of groundwater in the study area. In this aquifer, 2978 exploited wells were counted, accounting for 70.29% of the total number of wells, and the annual exploitation quantity is as high as 2.20 × 10^8^ m^3^, accounting for 65.43% of the total amount of exploitation in the area. Confined aquifer Ⅰ is the next most utilized source and has 973 wells, accounting for 22.96% of the total number of wells, and the annual exploitation quantity is 9.41 × 10^7^ m^3^, accounting for 27.93% of the total amount of exploitation. There are 286 wells and the exploitation quantity is 2.24 × 10^7^ m^3^ in confined aquifer III, accounting for 6.75% and 6.64% of the total number of wells and the annual total exploitation quantity, respectively.

From the perspective of utilization, the exploited deep groundwater is mainly used for industrial production, followed by domestic usage, and these factors account for 59% and 41% of the total, respectively.

#### 3.4.2. Geological Environmental Problem: Land Subsidence

**Figure 8:**
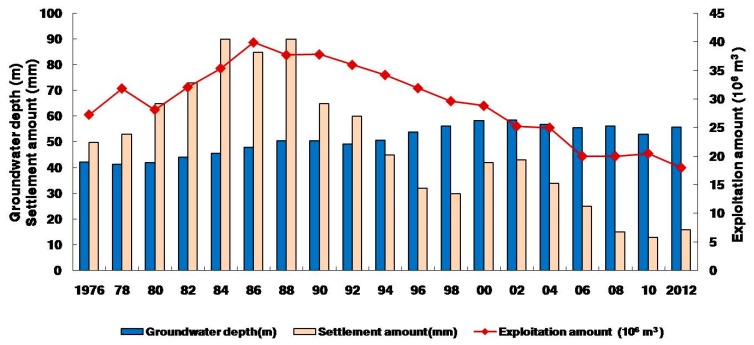
Relationship among groundwater exploitation amount, groundwater depth and settlement amount of land subsidence in the Su-Xi-Chang region.

Groundwater exploitation often leads to a variety of geological and environmental problems and land subsidence is the main one in the plain region. The occurrence and development of land subsidence in the Su-Xi-Chang region are closely related to groundwater exploitation and these factors are temporally and spatially consistent (Figure 8). Before the 1980s, groundwater exploitation was mainly concentrated in the three cities of Suzhou, Wuxi and Changzhou, and so land subsidence occurred in the central city areas. After the 1990s, large-scale exploitation of groundwater occurred and ground subsidence quickly spread across the entire study area. As the scale and intensity of the exploitation increased, the groundwater table continued to decline and the rate of land subsidence also increased correspondingly.

**Figure 9:**
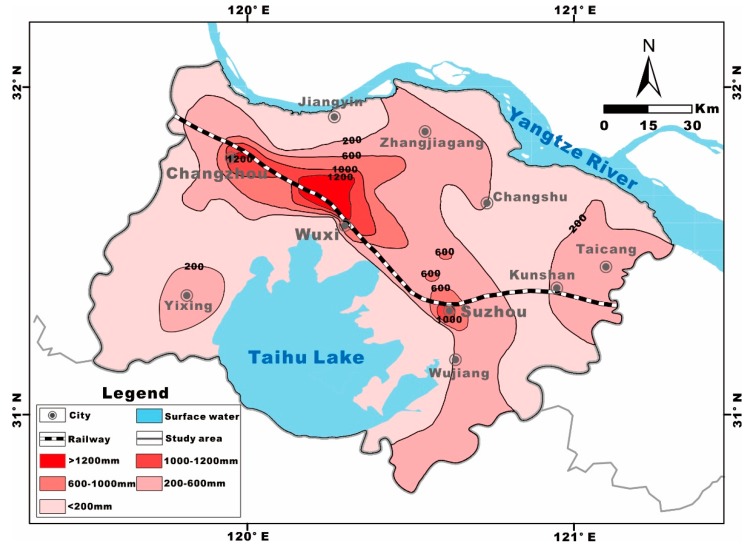
Land subsidence contour plot of cumulative settlement amount in the Su-Xi-Chang region (2015).

The area of cumulative settlement that is greater than 200 mm covers more than 5770 km^2^, accounting for approximately 40% of the total Su-Xi-Chang region. Moreover, the 600 mm contour has been conjoined in three central cities, occupying an area of more than 1500 km^2^ (Figure 9).

## 4. Conclusions

Higher values of TDS (224.89–1086.70 mg/L), turbidity (0.1–18.60 NTU) and nitrate (0.01–154.00 mg/L) were observed in some samples according to the China groundwater quality standards and WHO drinking water standards. Furthermore, extremely high ammonia concentrations were detected and the concentration of ammonia (0.01–32.90 mg/L) had a mean value of 0.72 mg/L, which exceeded the class II groundwater quality standards in China by 36 times. Weathering, dissolution of carbonate, halite and silicate, as well as cation exchange, were suggested as the main mechanisms controlling the hydrogeochemical signatures and processes in this region. Anthropogenic activities, such as agricultural practices, waste discharge, acid rain, land use and reclamation, accompanied by rock–water interactions, were mainly responsible for the increased concentrations of ammonia, total phosphorus, sulfates and nitrates. Constant assessment and continuous monitoring of groundwater are urgently needed to safeguard the health of the communities and to ensure sustainable development in the Su-Xi-Chang region.

## Figures and Tables

**Table 1:** Statistical summary of the physicochemical parameters of shallow groundwater in the Su-Xi-Chang region.

Parameter	Min.	Max.	Mean	QSGW ^a^	EQSSW ^b^	SDWQ ^c^	GDWQ ^d^
Temperature (°C)	11.00	22.50	16.89	n.s.	n.s.	n.s.	n.s.
EC (μS cm^−1^)	120.00	2850.00	1031.26	n.s.	n.s.	n.s.	n.s.
pH	5.59	7.82	7.00	6.5–8.5	6–9	6.5–8.5	6.5–8.5
TDS (mg L^−1^)	224.89	1086.70	616.69	≤500	n.s.	≤1000	≤1000
Turbidity (NTU)	0.1	18.60	3.85	≤3	n.s.	≤1	≤1
DO (mg O_2_ L^−1^)	0.03	5.51	0.97	n.s.	≥6	n.s.	n.s.
Ammonia (mg N L^−1^)	0.01	32.90	0.72	≤0.02	≤0.5	≤0.5	n.s.
TP (mg P L^−1^)	0.01	2.02	0.15	n.s.	≤0.1	n.s.	n.s.

n.s. not specified; ^a^ Quality standard for groundwater, class II (GB/T 14848-93 [47]); ^b^ Environmental quality standards for surface water, class II (GB 3838-2002 [50]); ^c^ Standards for drinking water quality (GB 5749-2006 [48]); ^d^ Guidelines for drinking-water quality [49].

**Table 2:** Statistical summary of the hydrogeochemical parameters of the shallow groundwater in the Su-Xi-Chang region.

Parameter	Min.	Max.	Mean	SD	Skewness	Kurtosis
Ca^2+^	26.20	162.00	90.09	28.93	0.26	−0.50
Mg^2+^	6.07	64.00	30.23	11.86	0.58	−0.07
Na^+^	8.32	159.00	72.63	29.23	0.63	0.05
Cl^−^	7.96	191.00	84.39	36.18	0.79	0.28
SO_4_^2−^	2.45	187.00	89.40	39.04	0.34	−0.17
HCO_3_^−^	113.00	706.00	353.70	132.67	0.64	−0.25
NO_3_^−^	0.01	154.00	22.07	26.85	2.04	4.73
H_2_SiO_3_	11.10	55.40	25.14	6.87	1.31	3.01

Units for all chemical indices are mg/L except pH, SD (standard derivation).

**Table 3:** Pearson correlation (r) for the hydrogeochemical parameters of the shallow groundwater in the Su-Xi-Chang region.

Parameter	pH	TDS	Ca^2+^	Mg^2+^	Na^+^	Cl^−^	SO_4_^2−^	HCO_3_^−^	NO_3_^−^	SiO_2_
pH	1	0.136 *	0.021	0.129	0.170 **	0.211 **	0.108	0.048	0.044	0.080
TDS	0.136 *	1	0.745 **	0.651 **	0.705 **	0.695 **	0.538 **	0.701 **	0.311 **	0.163 *
Ca^2+^	0.021	0.745 **	1	0.424 **	0.262 **	0.395 **	0.421 **	0.666 **	0.247 **	0.064
Mg^2+^	0.129	0.651 **	0.424 **	1	0.442 **	0.466 **	0.246 **	0.710 **	−0.019	−0.037
Na^+^	0.170 **	0.705 **	0.262 **	0.442 **	1	0.706 **	0.329 **	0.526 **	0.010	0.112
Cl^−^	0.211 **	0.695 **	0.395 **	0.466 **	0.706 **	1	0.345 **	0.311 **	0.109	0.109
SO_4_^2−^	0.108	0.538 **	0.421 **	0.246 **	0.329 **	0.345 **	1	0.008	0.343 **	−0.141 *
HCO_3_‑	0.048	0.701 **	0.666 **	0.710 **	0.526 **	0.311 **	0.008	1	−0.093	0.121
NO_3_^−^	0.044	0.311 **	0.247 **	−0.019	0.010	0.109	0.343 **	−0.093	1	0.005
H_2_SiO_3_	0.080	0.163 *	0.064	−0.037	0.112	0.109	−0.141 *	0.121	0.005	1

**: Correlation is significant at the 0.01 level (two-tailed); *: Correlation is significant at the 0.05 level (two-tailed).

**Table 4:** Rotated component matrix for hydrogeochemical parameters.

Parameter	Factors			Communality
	FA1	FA2	FA3	
TDS	0.880	0.409	0.120	0.956
Ca^2+^	0.682	0.340	−0.041	0.582
Mg^2+^	0.823	−0.076	−0.197	0.722
Na^+^	0.740	0.130	0.190	0.600
Cl^−^	0.676	0.297	0.198	0.584
SO_4_^2−^	0.305	0.756	−0.226	0.717
HCO_3_^−^	0.879	−0.234	0.008	0.828
NO_3_^−^	−0.052	0.820	0.089	0.683
H_2_SiO_3_	0.083	−0.057	0.948	0.908
Eigenvalues	3.796	1.697	1.087	-
% of Variance	42.175	18.853	12.078	-
Cumulative %	42.175	61.028	73.106	-

Extraction method: Principal component analysis. Rotation method: Varimax with Kaiser normalization.

**Table 5:** The percent of variance in a given hydrogeochemical parameter accounted for by each factor.

Factors	TDS	Ca^2+^	Mg^2+^	Na^+^	Cl^−^	SO_4_^2−^	HCO_3_^−^	NO_3_^−^	H_2_SiO_3_
FA1	0.81	0.80	0.94	0.91	0.78	0.13	0.93	0.00	0.01
FA2	0.18	0.20	0.01	0.03	0.15	0.80	0.07	0.98	0.00
FA3	0.02	0.00	0.05	0.06	0.07	0.07	0.00	0.01	0.99
